# Several strains, one disease: experimental investigation of *Vibrio aestuarianus* infection parameters in the Pacific oyster, *Crassostrea gigas*

**DOI:** 10.1186/s13567-017-0438-1

**Published:** 2017-05-26

**Authors:** Marie-Agnès Travers, Delphine Tourbiez, Leïla Parizadeh, Philippe Haffner, Angélique Kozic-Djellouli, Mohamed Aboubaker, Marcel Koken, Lionel Dégremont, Coralie Lupo

**Affiliations:** 1 IFREMER, SG2M-LGPMM, Laboratoire de Génétique et Pathologie des Mollusques Marins, Avenue de Mus de Loup, 17390 La Tremblade, France; 2 Interactions Hôtes-Pathogènes-Environnements (IHPE), UMR 5244, CNRS, IFREMER, Université de Perpignan Via Domitia, Université de Montpellier, 34095 Montpellier, France; 3 BP 1119, Djibouti, Djibouti; 4 LABOCEA-CNRS, 120 Avenue Alexis de Rochon, 29280 Plouzané, France

## Abstract

This study investigated oyster infection dynamics by different strains of *Vibrio aestuarianus* isolated before and after the apparent re-emergence of this pathogen observed in France in 2011. We conducted experiments to compare minimal infective dose, lethal dose 50 and bacterial shedding for six *V. aestuarianus* strains. Whatever the strain used, mortality was induced in juvenile oysters by intramuscular injection and reached 90–100% of mortality within 5 days. Moreover, bacterial shedding was comparable among strains and reached its maximum after 20 h (≈10 EXP5 bacteria/mL/animal). Similarly, our first estimations of lethal dose 50 were comparable among strains (minimal infective dose around 0.4 × 10EXP5 bacteria/mL and LD50 around 10EXP5 bacteria/mL) by using seawater containing freshly shed bacteria. These results indicate that, at least with these criteria, despite *V. aestuarianus* strains genetic diversity, the disease process is similar. The strains isolated after the apparent re-emergence of the bacteria in 2011, do not present a more acute virulence phenotype than the reference strains isolated between 2002 and 2007. Finally, our study provides original and noteworthy data indicating that infected oysters shed bacteria at a level above the threshold of LD50 a few days before they die, meaning that infection is expected to spread in a susceptible population.

**Electronic supplementary material:**

The online version of this article (doi:10.1186/s13567-017-0438-1) contains supplementary material, which is available to authorized users.

## Introduction

*Vibrio aestuarianus* is a gamma proteobacterium causing oyster disease in France since 2001 [1–3]. Since 2011, the recent increase in adult oyster mortalities points again to this pathogen as a major concern for aquaculture. This bacterium has thus far been isolated in France, Ireland and Scotland [1, 4, 5] in association with chronic mortalities, and without any reported mortalities in Spain and Italy [6–8]. As other vibrios, *V. aestuarianus* can colonize different niches and has been isolated episodically from seawater, plankton, sediment and regularly from diseased animals (oysters and fish) [3, 7, 9–11].

Different strains isolated from oysters in the years 2000, all present different virulence degrees (i.e. inducing different levels of oyster mortalities after intramuscular injection) [1, 3]. Genome-based analyses and phylogenetic studies revealed the existence of two clades being equivalent in terms of virulence and of recent/ancient strain distribution. The ecological significance of these two subclasses remains therefore still unknown [12].

In term of pathology, studies with reference strains (01/032 and 02/041) demonstrated that after injection, *V. aestuarianus* can be found in the oyster hemolymph, where it inhibits hemocyte adhesion and phagocytosis capacity, and proliferates abundantly [13, 14]. Some virulence factors were identified like the metalloprotease Vam or one regulating gene (*varS*) [12, 15].

In a more physiological setting, the bacteria can also be transmitted by horizontal waterborne transmission: in cohabitation experiments [16] or in cohabitation experiments coupling *V. aestuarianus* with another recognized oyster pathogen, *Vibrio tasmaniensis* [17]. To render detection and quantification easier, green fluorescent protein (GFP)-tagging of strains can be used [21]; an approach also used in the current study. All these studies demonstrated that in lab experiments, the bacteria can pass from one animal to another through the seawater.

Even if *V. aestuarianus*’ ecology and virulence mechanisms are more and more studied, little is known about the infection processes and propagation dynamics inside an oyster population. Many infection parameters such as the minimum infective dose or the bacterial shedding during the pathogenesis are still unknown. In this publication, we developed new experimental protocols to estimate these parameters to better understand *V. aestuarianus* infection dynamics. Moreover, different strains, isolated before or after the recent re-emergence of the bacteria, will be compared with regard to these characteristics.

## Materials and methods

### Bacteria: growth conditions, detection and quantification

Six *V. aestuarianus* strains were used in this study and are presented in Table 1. Bacteria were grown in Zobell Broth [peptone 4 g/L, yeast extract 1 g/L, Tris buffer 0.5 g/L adjusted to pH 7.4 in artificial sterile seawater (ASW), supplemented with kanamycine 100 µg/mL for the GFP-tagged strain] and stock cultures were stored at −80 °C in Zobell containing 15% glycerol (v/v). For injection procedures, bacteria were grown for 24 h at 22 °C before adjustment to concentrations ranging from 5 × 10^2^ to 5 × 10^8^ bacteria/mL in ASW. Bacteria purity and concentration were checked by plating on Zobell agar.

**Table 1:** **Strains used in this study**

Strain	Isolation/origin	Clade	Virulence	Reference
02/041 GFP	02/041	A	Highly virulent	[21]
02/041	2002, Oyster, Brittany, *V. aestuarianus* subsp. *francensis* reference strain	A	Highly virulent	[17]
02/092	2002, Oyster, Brittany, France	B	Highly virulent	[12]
07/115	2007, Oyster, Brittany, France	A	Moderately virulent	[12]
12/016	2012, Oyster, Charente-Maritime, France	A	Highly virulent	[12]
12/063	2012, Oyster, Brittany, France	B	Highly virulent	[12]

Their origin, classification in clades previously described [12], and virulence estimated by intramuscular injection are also specified.

*GFP* green fluorescent protein.

*Vibrio aestuarianus* DNA quantities in oyster tissues or seawater were estimated by qPCR [3] after DNA extraction following QiaAmp^®^ tissue kit procedures (Qiagen^®^) and dilutions for oyster tissue extracts to adjust total DNA concentration to 5 ng/µL. A standard curve was established applying “diluted genomes”.

Briefly, the standard was prepared using the relation between the concentration of DNA in the *V. aestuarianus* 02/041 and the theoretical number of genomes, calculated on the basis of the DNA mass divided by the genomic molecular weight for *V. aestuarianus* (2.77 × 10^9^ g/mol, based on a genome size of 4.20 Mbp for *V. aestuarianus* [12]). Assays were performed on MX3000P and MX3005P machines (Agilent^®^) with the Brillant III Ultrafast kit (Stratagene^®^) following the manufacturer’s instruction. Bacterial concentrations were also estimated by Flow Cytometry (Coulter Epics XL cytometer, Beckman^®^) on 10 000 events or after 5 min with a threshold fixed on FL1 fluorescence.

### Specific pathogen-free oysters

Batches of Pacific oyster *Crassostrea gigas* were produced in March and April 2013 at the Ifremer hatcheries in Argenton, France and La Tremblade, France, and in April 2015 in La Tremblade. Oysters were kept in experimental facilities from spawning to the experiments described below with UV-treated and filtered seawater and under biosecurity conditions to avoid contamination with major pathogens inducing oyster mortalities: the Ostreid herpesvirus (OsHV-1) and *V. aestuarianus*. Twelve oysters were screened for the detection of these pathogens by standard protocols [18, 19]. Briefly, after hemolymph sampling in the adductor muscle sinus with a syringe equipped with a needle (0.9 × 25 mm), pieces of 50 mg of mantle and gills were dissected. Dilutions of hemolymph were plated on Zobell agar (10^−1^, 10^−2^). DNA from gills and mantle was extracted using a QiAmp^®^ kit. Presence of OsHV-1 and *V. aestuarianus* DNA was detected by qPCR as described [18, 19].

### Bacterial shedding experiments (Additional file 1)

Experiments were performed under static (closed circuit) conditions in aerated seawater maintained at 22 °C during all experiments. Oysters (mean individual weight 12.1 and 13.2 g) were anaesthetized with MgCl_2_ at a concentration of 50 g/L in tap water. Each oyster was intramuscularly injected with 50–100 µL of a *V. aestuarianus* suspension and placed into 0.5 L of UV-treated seawater. In a first part, we tested the 02/041-GFP strain at different doses, in two experiments. A total of sixty oysters were placed individually in a beaker (ten oysters for each of the four tested injected doses: 5 × 10^6^, 5 × 10^7^ (tested twice) 1 × 10^8^ and 5 × 10^8^ bacteria/animal). In a second part, the six strains were compared. Three groups containing each 10 animals were infected together with 5 × 10^8^ bacteria/animal in a single tank. Water was daily sampled and bacterial concentrations were estimated by flow cytometry and qPCR as described above. Estimated shedding rates were expressed as Bacteria/mL/oyster/day.

### Minimum infectious dose experiments (Additional file 1)

Experiments were performed under static conditions in aerated seawater maintained at 20 °C during all tests. Contaminated seawater was produced by placing 20 injected oysters in 10 L of UV-treated, filtered seawater (intramuscular (IM) injection with 5 × 10^7^ bacteria/animal) for 18 h. Bacterial concentration was checked by flow cytometry for GFP-tagged bacteria and qPCR.

In a first experiment, groups of 30 oysters were immersed in contaminated seawaters in 3 L tanks containing the different strains (six strains) at 5 × 10^7^ bacteria/animal for 24 h before seawater renewal. In a second experiment, oysters were individually exposed in 300 mL beakers to six different dilutions of contaminated seawater produced with 02/041 strain. After 24 h of contact, contaminated seawaters were replaced by fresh UV-treated seawater. Finally, in a third experiment, oysters were individually exposed for 24 h to contaminated seawater containing the different strains (six strains) at different doses (three doses). Control seawater corresponds to seawater collected from tanks with artificial sterile seawater (ASW) injected-oysters. Oysters were monitored daily and dead or moribund animals were removed and frozen for subsequent analysis for the duration of the experiment (15 days). Survivors were sacrificed at the end of the experiment. Individual mantle and gill samples, or whole animals in case of survivors, were tested for the presence of *V. aestuarianus* DNA.

### Statistics

Differences in cumulated mortalities were compared by Chi squared analysis. Survival data were analyzed using Kaplan–Meier survivor functions and compared using the log-rank test. Kruskal–Wallis tests were applied to assess significant differences between the shedding of the different strains, as a normal distribution was not assumed on log-transformed bacterial concentrations (Shapiro–Wilk test). For all tests, the significance threshold was *p* < 0.05. All these statistical analyses were gathered using biostaTGV [20]. Finally, the LD50 was estimated from dose effect curves by non-linear regression curve fitting using GraphPad Prism software (San Diego, CA, USA) when enough data were available. For comparison of strains, doses bracketing the LD50 (i.e. inducing more or less than 50% mortalities) were used.

## Results

### Bacterial shedding experiments

**Figure 1:**
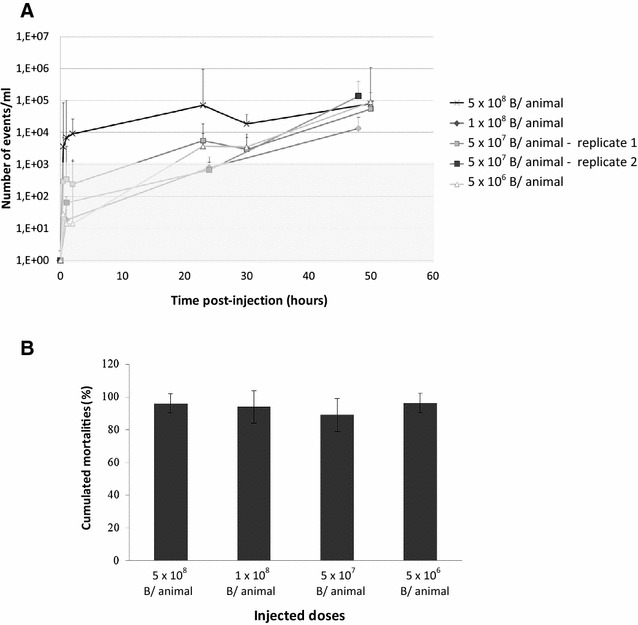
**Individual kinetics of shedding and mortalities observed after injection of different doses of** ***V. aestuarianus*** **02/041-GFP.** Experimental design is described in Additional file 1. **A** Kinetic of detection of bacteria in surrounding seawater after intramuscular injection of four doses of *V. aestuarianus* (5 × 10^6^ to 5 × 10^8^ bacteria/animal). Flow cytometry analyses of GFP-tagged bacteria detected in the surrounding seawater (3 measures per day and per condition). Quantification should not be considered below the 10^3^ events/mL (grey zone). **B** Cumulated mortalities recorded 5 days after the injection of different doses of *V. aestuarianus* 02/041-GFP (5 × 10^6^ to 5 × 10^8^ bacteria/animal). Experiments were realized twice over time with 3 replicates. Error bars correspond to standard deviation SD.

To determine the optimal dose to inject, and the appropriate time to measure shedding, an individual bacterial-shedding protocol was developed first on the *V. aestuarianus* reference strain 02/041 and its GFP counterpart. Daily individual bacterial shedding, corresponding to more than 10^3^ bacteria/mL/day, was first detected by qPCR and flow cytometry at 1 h post-challenge for 5 × 10^8^ injected bacteria, after 22 h for 5 × 10^7^ or 1 × 10^8^ injected bacteria, and after 30 h for 5 × 10^6^ injected bacteria (Figure 1A). Bacterial concentration reached its maximum of around 10^5^ bacteria/mL at 22 h post-infection with the 5 × 10^8^ bacteria injection, and of around 48 h for the 5 × 10^6^ and 5 × 10^7^ bacteria injected trials (Figure 1A). Cumulated mortality in intramuscularly injected oysters reached 90% for the 10-day experiment whatever the dose we used (Figure 1B). Neither mortality nor any *V. aestuarianus* DNA were observed in the control tanks.

**Figure 2:**
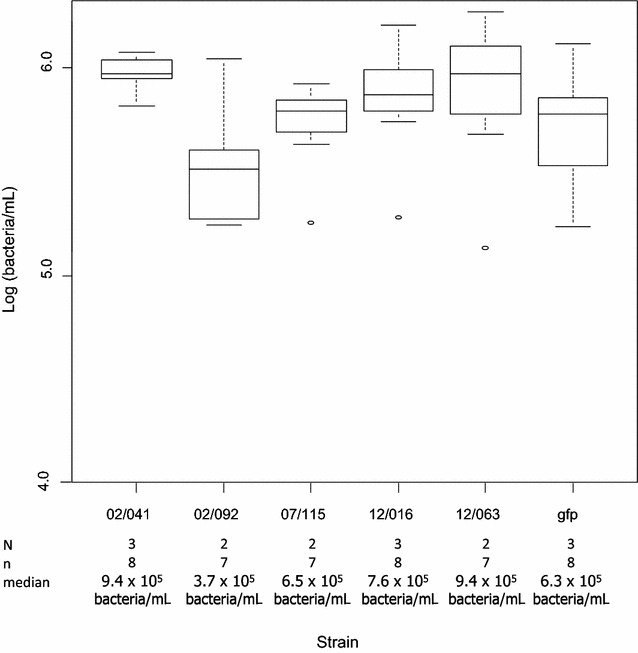
**Estimation of** ***V. aestuarianus*** **concentrations in the seawater by qPCR (Log[bacteria/mL]), 19** **h after intramuscular injection at DO**_**600nm**_ **=** **1 (approximately 5** **×** **10** ^**8**^ **bacteria/animal).** *V. aestuarianus* injected strains, number of independent experiments (N) or tanks (n), and median are specified. Error bars correspond to standard deviation SD. Experimental design is described in Additional file 1.

To evaluate the total shedding of the different *V. aestuarianus* strains, on a group of oysters, total DNA in the seawater was estimated by qPCR on samples taken between 19 and 22 h post-intramuscular injection of fresh concentrated bacterial suspensions (OD_600nm_ = 1) in three independent experiments. Between 3.7 × 10^5^ and 9.4 × 10^5^ bacteria/mL/animal were detected into the surrounding seawater, even for the moderately virulent strain (Figure 2). No statistically significant differences could be detected between strains (*p* = 0.116, Kruskal–Wallis test) nor between experiments (*p* = 0.412). Finally, for the GFP-tagged strain, quantification by flow cytometry (mean 2.3 × 10^5^ bacteria/mL; sd 6 × 10^4^) was considered as not significantly different from estimation obtained by QPCR (Figure 2; *p* = 0.324).

### Minimum infectious dose experiments

**Figure 3:**
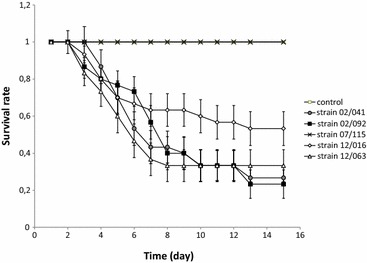
**Survival rates after immersion into undiluted contaminated seawater prepared with one of the five** ***V. aestuarianus*** **strains determined on 30 oysters (triplicates of 10 oysters placed in tanks).** Mortalities were checked daily. Two independent experiments were realized. Control oysters = crosses. Error bars correspond to standard deviation SD. Experimental design is described in Additional file 1.

To assess whether these shed *V. aestuarianus* can infect (enter into the host’s tissues) and to determine a minimal infective dose, sentinel oysters were placed into the contaminated seawater (undiluted or diluted). First mortalities were observed 4 days post infection by immersion into undiluted contaminated seawater, and recorded until day 13 (minimal average survival 23%, Figure 3). All the highly virulent strains tested (02/041, 02/092, 12/016, 12/063) induced comparable mortality kinetics, as revealed by Kaplan–Meier analyses (data not shown). It is important to notice that the moderately virulent strain 07/115 did not induce any mortality in sentinel oysters with similar amounts of bacteria. Finally, in control tanks, no mortality was noticed (Figure 3).

The average cumulated mortality at different contaminated seawater dilutions (for the reference strain 02/041) is displayed in Table 2. At 7.9 × 10^5^ bacteria/mL, mortality reached 70%, whilst at 0.5 × 10^5^ bacteria/mL, only 30% of mortality was observed. Mortality was not evidenced after immersion with contaminated seawater containing 3 × 10^4^ bacteria/mL or less. qPCR analyses of sampled moribunds indicated that all mortalities at all doses were linked to very large amounts of *V. aestuarianus* (more than 10^7^ bacteria/25 ng of total DNA).

**Table 2:** **Induced mortality after 24** **h of immersion in seawater contaminated with different concentrations of** ***V. aestuarianus*** **02/041 on oyster batch 1 (N** **=** **70, individual beakers)**

Bacteria/mL	Log bacteria/mL	Induced mortality (%)	Experiment	First day of mortalities
0.3 × 10^4^	3.40	0	1	/
0.8 × 10^4^	3.90	0	2	/
3 × 10^4^	4.46	0	1	/
0.5 × 10^5^	4.70	30	2	5
5.1 × 10^5^	5.33	60	1	4
7.9 × 10^5^	5.90	70	2	4

Oyster mortalities were checked daily. Experimental design is described in Additional file 1.

A lethal dose 50 (LD50), i.e. a dose inducing 50% mortality at the end of the experiment, can be estimated at 1.3 × 10^5^ bacteria/mL [i.e. 5.11 Log(bacteria/mL)] on this oyster batch, after an initial 24 h contamination by immersion in undiluted contaminated seawater at 20 °C (Table 2). Twelve surviving individuals (sampled at day 15, on the 60 initially infected animals) were crushed in their totality before analyses of *V. aestuarianus* content. In none of them bacterial DNA could be amplified by qPCR even if we cannot exclude the presence of small amounts of DNA with the actual detection limit of our diagnostic tool (1.6 × 10^2^ cells/mL [19]).

Lethal dose 50 was also estimated for the other strains to compare their virulence, on a second oyster batch by exposing the oysters to different doses of shed bacteria in individual beakers. Doses inducing 50% of mortality for the different strains were all estimated within 10^5^ and 10^6^ B/mL (Table 3). Interestingly, on this second oyster batch, LD50 for the 02/041 strain could be estimated between 5.8 × 10^5^ and 1.7 × 10^6^ bacteria/mL, which is higher than our first estimation (LD50 estimated at 1.3 × 10^5^ bacteria/mL on the first oyster batch), underlining the highly variable oyster physiology.

**Table 3:** **Doses of bacteria (bacteria/mL) in contaminated seawater inducing more or less than 50% of mortality after 24** **h of immersion of 10 oysters per condition in individuals beakers (oyster batch 2)**

Strains	Dose inducing *less* than 50% mortality	Dose inducing *more* than 50% mortality
12/016	7.5 × 10^4^ − 2.82 × 10^5^	1.27 × 10^6^
02/041	6.7 × 10^4^ − 5.75 × 10^5^	1.70 × 10^6^
12/063	2.4 × 10^4^ − 3.90 × 10^5^	2.26 × 10^6^
02/092	5.3 × 10^4^ − 1.34 × 10^5^	0.61 × 10^6^
07/115	1.48 × 10^6^	n.d.

Different strains of *V. aestuarianus* (02/041-GFP, 02/041, 02/092, 07/115, 12/016, 12/063) were used to produce contaminated seawaters: source oysters were intramuscularly injected with 5 × 10^7^ bacteria/animal. After 18 h, contaminated seawaters were serially diluted in fresh UV-treated seawater, and bacterial concentration was estimated by QPCR. Experimental design is described in Additional file 1.

## Discussion

This study provides first insights into *V. aestuarianus* infection dynamics by conducting experimental infections to quantify bacterial shedding from infected oysters over time and to determine a minimal infective dose and an LD50. Moreover, the different strains were compared for properties other than and complementary to the classically “rate of mortality after intramuscular injection” [12].

To investigate bacterial shedding from oysters, animals were intramuscularly injected with different doses of bacteria, to ensure that the animals were synchronously infected with a standardized amount of bacteria. Bacteria released into the surrounding seawater were quantified by qPCR and flow cytometry using a GFP-tagged strain, previously demonstrated as comparable to the wild-type strain in terms of induced mortality. Even if GFP-tagged bacteria allow a real-time monitoring of bacteria in suspension, such tools present a moderate sensitivity, previously estimated for this strain around 10^3^ cells/mL [21]. On the other side, classical qPCR allows the detection of as little as 10^2^ cells/mL [19] but does not distinguish dead bacteria from living ones. By combining these tools, a quick shedding was observed leading to detection, as soon as 1 h post infection of bacteria reaching 10^3^ to 10^5^ cells/mL/animal depending on the injected dose.

Progressive accumulation of bacteria was observed in each tank, reaching a plateau after 24 h for the highest dose and 50 h for the lowest one. It is noticeable that animal death was not observed until 50 h, which corresponds to the time limit of detection. These results are consistent with other models, in which moribund animals are well-known as important shedders, via thus far still unknown mechanisms. For instance, in the *Aeromonas salmonicida*–*Salmo salar* model, release of bacteria from morbid animals was estimated in the order of 10^5^ to 10^8^ cfu/fish/h [22]. For the bacteria *Vibrio harveyi* infecting the abalone *Haliotis tuberculata*, bacterial shedding was estimated around 10^4^ bacteria/abalone/mL [23].

Even if *V. aestuarianus* strains can present genetic diversity, our results suggest that the disease process is similar: comparison of the release of and the impact of different strains isolated in 2002 and 2012, and belonging to clade A or B [12] did not show noticeable difference. Similar shedding characteristics were observed in the different strains. No remarkable difference of bacterial shedding for the strains from clade A (12/016 or 02/041) or clade B (12/063, 02/092), and no changes in induced mortalities of sentinel oysters could be detected. In all moribund oysters, large amounts of *V. aestuarianus* were observed after hemolymph plating (more than 10^7^ bacteria/mL), confirming the colonization and multiplication of the pathogen inside the animal.

Cohabitation protocols and immersion protocols in the presence of sediment were already described for this bacterium [11, 17]. However, in our experiments, cohabitation and contamination with cultured bacteria appeared less reproducible (Travers, pers. comm.) than experimental infections with oyster-shedded bacteria, as if the passage through the host homogenized or induced bacterial infectivity. Similarly, studies of the interaction of *Vibrio vulnificus* with eels suggest that host cell contact is required for its pathogenicity [24].

This protocol was chosen to estimate bacterial minimal infective doses and LD50s as it most closely mimics natural exposure to a pathogen [25]. Infections, duplicated in time, with different doses of shedded bacteria were performed either on ten separated individuals (10 tanks) or on thirty animals in triplicates (three tanks). With 5 to 8 × 10^5^ bacteria/mL, cumulated mortalities reached in each case 77% in 15 days. However, to limit bacterial exposure to the first 24 h and to avoid potential transmission between animals in a same tank, final LD50 and minimal infective doses were estimated on animals physically separated in ten 0.5 L-tanks. The minimum infective dose required to reliably induce infection after a 24 h immersion period in contaminated seawater, was estimated at 0.4 × 10^5^ bacteria/mL and the LD50 around 10^5^ bacteria/mL. Comparison of the doses inducing 50% of mortality on two oyster batches revealed some differences (for the 02/041 strain) even if oysters were comparable in term of age and size. Genetic background, as well as life history traits can influence oysters sensitivity to pathogens (e.g. effective infectious dose, and survival [16, 26, 27]). This sensitivity may also be linked to other transmission parameters (e.g. shedding). In absence of standardized oyster lineages, we should thus favor (1) experiments in which all epidemiological parameters are recorded in parallel on the same animals and (2) experiments that are repeated on different oyster batches.

Interestingly, taken together, our results indicate that one contaminated oyster placed into 350 mL of seawater can release 5 to 10 × 10^5^ bacteria/mL in 24 h, and the LD50 was estimated at 1 × 10^5^ bacteria/mL. These findings are very significant for the dynamic of the disease because they indicate that shedding begins at a level already above the LD50 before mortalities occur, meaning that an infected oyster can pass the infection to at least one other oyster, clearly favoring disease-spread in a susceptible population. Additionally, by intramuscular injection with the same bacterial isolate (02/041), as little as 100 bacteria/animal can induce more than 80% mortalities [12]. These data highlight the importance of the initial step in the infection process. Moreover, analyses of surviving individuals revealed the absence of bacteria, which might suggest that these animals were not infected during the 24 h-bath or cleared the infection. This observation may be linked to genetic/physiological specificities of these animals. Even if the heritability for survival of *C. gigas*, when exposed to *V. aestuarianus* is low to moderate [28], oysters selected for their higher resistance to *V. aestuarianus* infection could constitute a future line of research. The basis of the observed resistance may rely on mechanisms developed by the oysters to prevent the bacteria to enter and/or to multiply. However, more sensitive tools and more targeted screening of tissue are needed to confirm this hypothesis. Future efforts on the comprehension of this crucial initial step are thus necessary. First studies suggested that *V. aestuarianus* could be found after a few hours in the hemolymph and mantle during cohabitation challenges [17, 21]. However the entry site, the targeted tissues, and the timeline are still unknown.

*Vibrio aestuarianus* cells are difficult to find and culture from environmental samples. Moreover, only little field data on *V. aestuarianus* concentrations in oyster environments during epidemics are available. Recent intensive field surveys allowed its isolation from oysters, mussels, plankton, sediments and seawater in France, Spain and Italy [6, 7, 11]. *V. aestuarianus* can reach 10^3^ to 10^4^ cell/g sediment in warm months [7, 11] and 10^2^ cell/mL in the seawater column in the Adriatic sea. Interestingly, *V. aestuarianus* can be found associated with plankton where up to 10^6^ bacteria/g were quantified [7]. However, detection protocols [19] did not allow the discrimination of virulent and non-virulent strains, and thus the importance of the plankton compartment in the effective concentration of virulent *V. aestuarianus*, and/or its transmission to oysters is still unknown.

Finally, our study demonstrates that virulence classification determined only through injection protocols with high doses of bacteria can be source of debates, leading some authors to consider some strains as pathogens [29]. The *V. aestuarianus* 07/115 strain, induces high levels of mortality when injected at 5 × 10^8^ bacteria/animal, but does not affect oysters when injected at very low doses (10^2^ bacteria/animal [12]) or when present in high concentrations in the surrounding water. Future efforts on animal models and infection protocols are certainly needed to clearly define mollusk pathogens.

In conclusion, this study provides new experimentally acquired data on the *V. aestuarianus*–*C. gigas* interaction by estimating bacterial shedding and bacterial infective doses. However, more integrative population-based studies are now needed to try to link these first parameter estimations to natural occurring epidemics.

## Additional file

**Additional file 1.** **Experimental set-up.** Objective of the different experiments, as well as strains, doses, number of oyster, design and monitoring are indicated.
