# Supplementary material for: Several strains, one disease: experimental investigation of Vibrio aestuarianus infection parameters in the Pacific oyster, Crassostrea gigas
Source: Vet Res. 2017 May 26;48:32. doi: 10.1186/s13567-017-0438-1 (PMC5446674; doi:10.1186/s13567-017-0438-1)
Supplement: Supplementary file 1 — Additional file 1. Experimental set-up. Objective of the different experiments, as well as strains, doses, number of oyster, design and monitoring are indicated. [file 13567_2017_438_MOESM1_ESM.pdf]

	Bacterial shedding experiments		Infectious doses experiments		
	**Part I**	**Part II**	**Part I**	**Part II**	**Part III**
Objective	Individual estimation of bacterial shedding over time	Global estimation of bacterial shedding for different strains	Estimation of survival in presence of different freshly shedded strains	Accurate estimation of LD50 for our reference strain	Global estimation of doses inducing 50% of mortality for different freshly shedded strains
Strains	02/041-GFP	02/041-GFP, 02/041, 02/092, 07/115, 12/016, 12/063	02/041-GFP, 02/041, 02/092, 07/115, 12/016, 12/063	02/041	02/041, 02/092, 07/115, 12/016, 12/063
Dose	4 doses (from $5x10^6$ to $5x10^8$ bacteria/animal) + 1 control	1 dose ($5x10^8$ bacteria/animal)	1 dose ($1x10^5$ bacteria/ml)	6 doses ($3x10^3$ to $8x10^5$ bacteria/ml)+ 1 control	3 doses (around 10^4 to 10^6 bacteria/ml) + 1 control
Design	Injection of oysters placed in individual beakers<br><br>Realised twice for 1 dose	Injection of oysters placed in tanks (triplicate of 10 animals)<br><br>Temporal replicates (2 or 3 times) were realised	1) Production of contaminated seawater by injecting oysters. Adjustment of bacterial load<br><br>2) Immersion for 24h of sentinelle oysters (triplicate of 10 animal) into undiluted contaminated seawaters.	1) Production of contaminated seawater by injecting oysters. Dilution of the contaminated seawater.<br><br>2) Immersion for 24h of sentinelle oysters (10 oysters per dose placed in individual beakers).	1) Production of contaminated seawater by injecting oysters. Dilution of the contaminated seawater.<br><br>2) Immersion for 24h of sentinelle oysters (10 oysters per dose placed in individual beakers).
Number of oysters	60 oysters	540 oysters	350 oysters<br><br>Sources : 20 per strain + control<br><br>Sentinelles : 30 per strain + control	90 oysters<br><br>Sources : 20 + control<br><br>Sentinelles : 10 per dose + control	280 oysters<br><br>Sources : 20 per strain + control<br><br>Sentinelles : 10 per dose per strain + control
Monitoring	Sampling of seawater at different timepoints (0; 0.5; 1; 2; 23; 30; 50 hours), and mortality recording<br>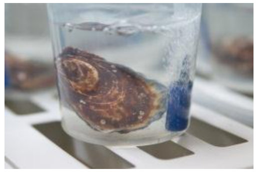	Sampling of seawater at 19h post-injection, and mortality recording<br>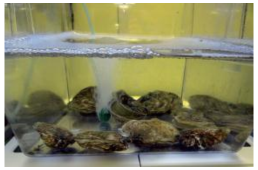	Mortality recording and analyses of moribund animals<br>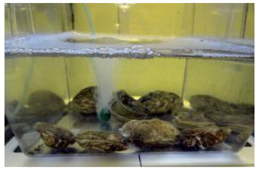	Mortality recording and analyses of moribund animals<br>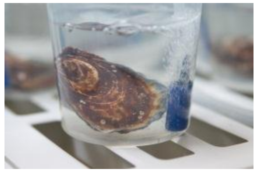	Mortality recording and analyses of moribund animals<br>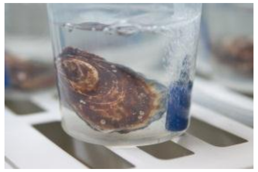
